# ﻿Heathers (*Erica*, Ericaceae) of Madagascar: taxonomy, evolution, ecology and uses

**DOI:** 10.3897/phytokeys.256.141375

**Published:** 2025-05-20

**Authors:** Jan Hackel, Andriantsilavo H. I. Razafimanantsoa, Vincent Porcher, Michael D. Pirie

**Affiliations:** 1 Department of Ecology, Environment and Plant Sciences (DEEP), Stockholm University, Stockholm, Sweden Stockholm University Stockholm Sweden; 2 Department of Biology, Philipp University of Marburg, Marburg, Germany Philipp University of Marburg Marburg Germany; 3 Human Evolution Research Institute, Department of Geological Sciences, University of Cape Town, Cape Town, South Africa University of Cape Town Cape Town South Africa; 4 CEFE, University of Montpellier, CNRS, EPHE, IRD, Montpellier, France University of Montpellier Montpellier France; 5 University Museum, University of Bergen, Postboks 7800, NO-5020 Bergen, Norway University of Bergen Bergen Norway

**Keywords:** *anjavidy*, *Erica*, ericoid thicket, fire ecology, Madagascar, Mascarenes, *Philippia*, vegetation history

## Abstract

The plant genus *Erica* L. (heathers; *anjavidy* in Malagasy) has 35 recognised species in Madagascar, but there has not been a taxonomic revision since 1927 and there are few identification resources. We review available data for Malagasy *Erica* (previously treated as *Philippia*), summarise diagnostic species descriptions and incorporate them into the *Erica* Identification Aid. There is clearly variation in current species concepts that requires further study. Malagasy *Erica* most likely represent a single clade also encompassing species from the Mascarenes, but resolution is poor and most species remain unsequenced. *Erica* is found in several of Madagascar’s ecosystems, including the high-altitude “ericoid thickets” where diversity is highest, but it is absent from the extensive dry western areas. Habitats include the ericoid thickets, shrubland–grassland mosaics in the central highlands and on the eastern coast, and *Uapacabojeri* (tapia) savanna. Many *Erica* species are likely to be part of dynamic ecosystems with infrequent fire regimes. The palaeorecord indicates a more widespread ericoid shrub vegetation during the last glacial period. There may be both wind- and insect-pollinated species. *Erica* is mainly used as fuelwood in Madagascar, but local uses as tools and medicine have also been reported. Estimates suggest at least one-fifth of the species may be threatened, but formal assessments are lacking. Taxonomic revision of the group, coupled with phylogenomic, ecological and ethnobotanic studies, is an urgent priority.

## ﻿Introduction

The huge plant genus *Erica* L. (heathers, Ericaceae) has one of its regional centres of diversity in Madagascar. Currently, 35 species are accepted for the island, all endemic (Antonelli et al. 2022; Elliott et al. 2024; World Flora Online 2024). *Erica* is one of three Ericaceae genera in Madagascar along with *Agarista* D.Don, with seven species, and *Vaccinium* L., with four species. Heathers and other Ericaceae are key to understanding Malagasy vegetation dynamics: They form one of the main components of a globally distinctive and endangered high-mountain vegetation type, “Madagascar ericoid thickets” (Crowley 2004; Dinerstein et al. 2017). They also occur in other vegetation types especially in the central highlands of Madagascar, where the role of humans and fire in shaping open habitats is debated (e.g. Joseph et al. 2021; Silander et al. 2024). According to Phelps et al. (in press), “the genus *Erica* is likely key to understanding grassland–shrubland–savanna dynamics” in Madagascar. Yet there has not been a taxonomic revision since that of Perrier de la Bâthie (1927b). The genus and family were not treated in two major reference works on the natural history of Madagascar (Goodman and Benstead 2004; Goodman 2022), nor in the *Flore de Madagascar et des Comores* series. Increasing our taxonomic and ecological knowledge of Malagasy *Erica* is thus a priority for the conservation of Madagascar’s highly endangered biodiversity (Ralimanana et al. 2022), including through the work of the Global Conservation Consortium for *Erica* (Pirie et al. 2022). Here, we summarise existing data on *Erica* in Madagascar to identify knowledge gaps and research priorities.

## ﻿Taxonomy

*Erica* (Ericoideae, Ericeae) is easily distinguished from *Agarista* and *Vaccinium* (both Vaccinioideae), most obviously by its often needle- or scale-like leaves characterised by revolute margins almost touching on the underside (“ericoid leaves”) and arranged in whorls of 3–6, and its persistent corolla enveloping the fruit (Oliver 2000; Schatz 2005). However, within *Erica* and the tribe Ericeae, generic limits were a longstanding subject of debate that was only resolved towards the end of the last century.

**Figure 1.**
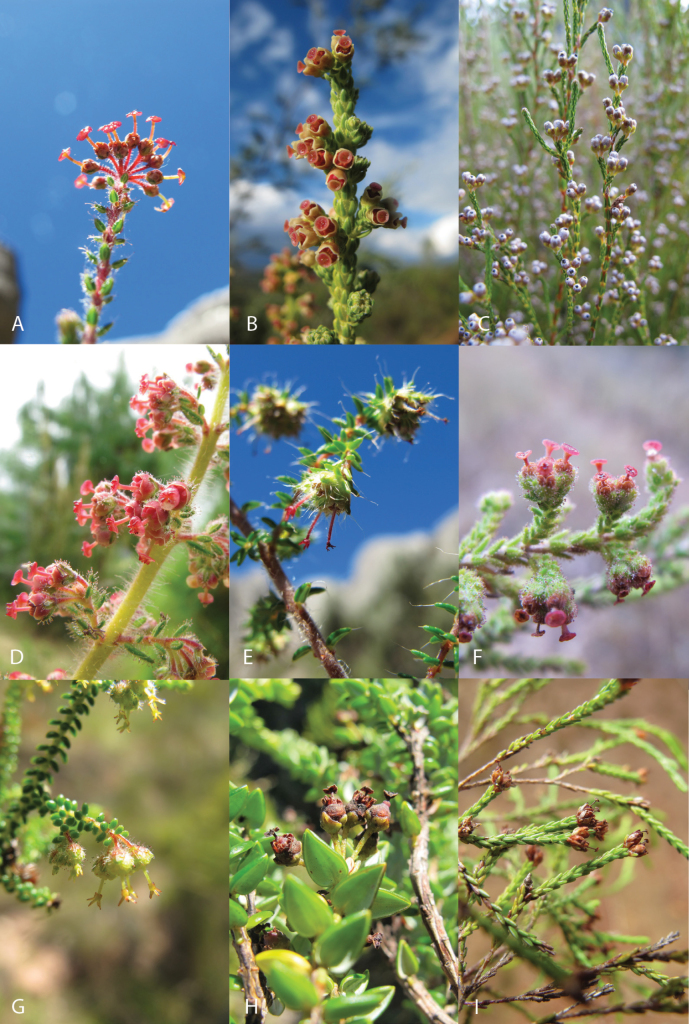
Morphological diversity of *Erica* in Madagascar: a selection of specimens collected by E.G.H. Oliver and colleagues **A***E.bojeri* (EO12649) **B***E.boutonii* Dorr & E.G.H.Oliv. (EO12676) **C***E.cryptoclada* (Baker) Dorr & E.G.H.Oliv. (EO12642) **D***E.hebeclada* Dorr & E.G.H.Oliv. (EO12690) **E***E.humbertii* (H.Perrier) Dorr & E.G.H.Oliv. (EO12648) **F***E.myriadenia* (Baker) Dorr & E.G.H.Oliv. (EO12677) **G***E.parkeri* (EO12626) **H***E.perrieri* (EO12635) **I***E.rakotozafyana* Dorr & E.G.H.Oliv. (EO12681). Photos: Michael D. Pirie.

The current state of taxonomy in Malagasy *Erica* (morphological diversity illustrated in Fig. 1) is largely founded on the work of Perrier de la Bâthie (1927a, 1927b, 1930, 1934). His revision (Perrier de la Bâthie 1927b) in particular already included descriptions of most of the species recognised today, sixteen of which were new. Perrier treated them all under the African-Malagasy genus *Philippia*, distinguished by small flowers with a zygomorphic calyx lacking, or resulting from the fusion of, the subtending bracts typical of *Erica* in the strict sense. Both Perrier and Alm and Fries, who published a monograph of *Philippia* earlier in the same year (Alm and Fries 1927), wrote of inconsistency in the characters distinguishing genera in Ericeae. The latter erected a separate genus, *Mitrastylus* Alm & T.C.E.Fr., for two species with a distinctive folded-back stigmatic disc (a structure somewhat resembling a broken umbrella with rib-like lobes, in contrast to the expanded disc form more typical of wind-pollinated *Erica*). Both species of *Mitrastylus* were from Madagascar: *E.parkeri* (Baker) Dorr & E.G.H.Oliv. (Fig. 1G) and *E.madagascariensis* (H.Perrier) Dorr & E.G.H.Oliv. All the taxa originally described under *Philippia* or treated under other genera in Ericeae (including *Mitrastylus*) were later transferred into *Erica* with new combinations and names by Dorr and Oliver (1999). This followed Oliver (1987, 1988) whose work extended to including in a broadly defined *Erica* all related so-called ‘minor genera’ (Nelson et al. 2024). After Perrier’s revision (1927), three additional Malagasy species and one subspecies were described: *Ph.quadratiflora* H. Perrier (= *E.quadratiflora* (H. Perrier) Dorr & E.G.H. Oliv.) in Perrier de la Bâthie (1930); *Ericamarojejyensis* Dorr, *Ericabosseri* Dorr, and Ericalecomteisubsp.ravinakely Dorr in Dorr and Oliver (1999). There has not been a more recent revision.

Perrier de la Bâthie (1927a) grouped the Malagasy species according to morphological characters which he ranked in order of importance. Most important was the shape of the stigma, on which Perrier initially defined two sections: I (Cornigerae) for species with expanded stigmatic lobes (including, but not limited to, the ‘mitrastylous’ species; his numbers 1–7, Appendix 1); and II (Discoidales) for those with a more typical discoid stigma (numbers 8–34). He later (Perrier de la Bâthie 1930) rejected the separate genus *Mitrastylus* described by Alm and Fries (1927), on the basis of the continuum in stigma forms, and abandoned this sectional division. However, he effectively reconfirmed his concepts of similarity between species, defining six groups that were largely consistent with the sequence of taxa presented in Perrier de la Bâthie (1927a) (Appendix 1). The other characters he cited in his main revision (Perrier de la Bâthie 1927a) were, in descending order of importance: the arrangement of the leaves (particularly in whorls of three versus of four); indument (he distinguished four types differing e.g. by length and presence or not of glands); leaf shape; inflorescence structure (terminal clusters of flowers, or not); flower and style shape (particularly shape and relative size of sepals); and degree of fusion of the stamens. Some species are distinct and easily identified even from images, such as *E.perrieri* Dorr & E.G.H.Oliv. with its unique open-backed leaves (Fig. 1H). Most of the others are less obvious.

Perrier de la Bâthie (1927a) noted the considerable variation given age, season, and conditions (including the impact of fire), with particular emphasis on habit and on changes in (or loss of) diagnostic characters after flowering. Most specimens available to him were in fruit, with very few collected at flowering time (Perrier de la Bâthie 1930). He also mooted hybridisation between species. His concepts for some particularly variable species (such as *E.goudotiana* (Klotzsch) Dorr & E.G.H. Oliv., treated as *Philippiacauliflora* Hochr.) deliberately focused on the most common form, with deviation from that form described separately. Dorr and Oliver (1999) also indicated variation within species e.g. within *E.goudotiana*, for which they noted that formal recognition ‘awaits further study’. The numbers of specimens available for such study appears to be small given the numbers of taxa involved, and a large proportion of that which is documented in available archives remains unidentified (see below). The species were included in earlier versions of the *Erica* Identification Aid (*Erica* ID aid; Oliver et al. 2024), but the data was largely incomplete, preventing its effective use. There may be undescribed variation within the current backlog of unidentified material. We need tools to facilitate identifications of Malagasy *Erica* species, and more collections to better understand the complex patterns of morphological variation in the group.

In this work we summarised information available from the literature to populate the ID aid. Through work on the World Flora Online (WFO; Borsch et al. 2020) Taxonomic Expert Network for Ericaceae, we have made openly available the current state of knowledge of *Erica* nomenclature (Elliott et al. 2024). A list of currently accepted Malagasy taxa with links to WFO and GBIF and diagnostic descriptions derived from Perrier de la Bâthie (1927a) and Dorr and Oliver (1999) is presented in Appendix 1. We have incorporated these into an update of the *Erica* ID aid (current version: https://doi.org/10.5281/zenodo.10407033), including coding of characters used in the ID aid for narrowing down potential identifications.

## ﻿Phylogenetics and evolution

**Figure 2.**
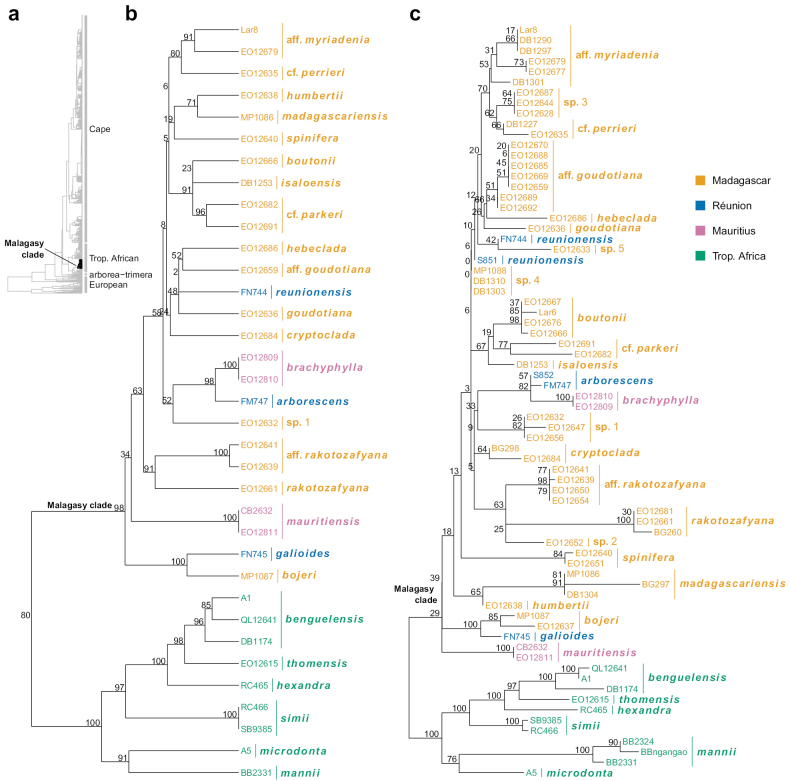
Phylogenetic relationships of Malagasy *Erica***a** position of the Malagasy clade in the genus according to Pirie et al. (2024). The tree includes 771 accessions and several nuclear and plastid markers **b** focus on the Malagasy clade and its Tropical African sister clade (same tree as in a) **c** tree estimated from the same marker set as in a-b but expanded with ITS for 36 additional accessions. Values on nodes are Felsenstein bootstrap support percentages.

Malagasy *Erica* form a single clade which also includes species from the Mascarenes (Fig. 2), based on the available phylogenetic sampling. A single origin of the Malagasy *Erica* was already postulated by Perrier de la Bâthie (1927a) and mirrors numerous other plant and animal radiations that occurred following dispersals to the island (Vences et al. 2009). Molecular phylogenetic studies based on regions from the chloroplast genome and nuclear ribosomal DNA (including the internal transcribed spacer region, ITS), have supported this Malagasy clade and shown it to be nested within a broader tropical African clade, which itself is sister to the large Cape radiation of *Erica* (Fig. 1A, B; Pirie et al. 2011, 2016, 2024). We here expanded the dataset of Pirie et al. (2024) with ITS data for additional collections from Madagascar and Mascarene islands, bringing the totals to 52 from Madagascar (including 13 identified species represented by 22 collections and 30 further collections unidentified to species), four from Mauritius (two collections each of both species), and five from Réunion (representing all three species) (Suppl. material 2). We estimated a Maximum Likelihood phylogeny for the Malagasy clade and its sister group, using RAxML v.8 (Stamatakis 2014) and otherwise following the methods of Pirie et al. (2024). The results are presented in Fig. 2, along with those of Pirie et al. (2024).

The topology presented by Pirie et al. (2024) showed several supported clades. *Erica* species from the Mascarenes, i.e. Mauritius (*E.brachyphylla* (Benth.) E.G.H.Oliv., *E.mauritiensis* E.G.H.Oliv.) and Réunion (*E.arborescens* (Willd.) E.G.H.Oliv., *E.galioides* Lam., *E.reunionensis* E.G.H.Oliv.), form at least four separate lineages within the Malagasy clade. This strongly suggests that *Erica* dispersed once from continental Africa to Madagascar, diversified there and then further dispersed several times independently to the Mascarenes. Neither *Ericacomorensis* (Engl.) Dorr & E.G.H.Oliv. (Comoros archipelago) nor *E.mafiensis* (Engl.) Dorr (Mafia island, off the Tanzanian coast) have yet been sequenced: Whether these also belong to the Malagasy clade warrants testing.

The close relationship of *E.madagascariensis* and *E.humbertii* (H.Perrier) Dorr & E.G.H.Oliv. might have been expected from Perrier de la Bâthie’s (1927a) revision, which grouped these mitrastylous species together. However, *E.parkeri*, which also shares this trait, falls in a separate clade with another (unidentified) mitrastylous species plus *E.isaloensis* (H.Perrier) Dorr & E.G.H.Oliv. and *E.boutonii*. This warrants further investigation, suggesting that important diagnostic characters potentially relating to pollination mode – including the mitrastylous stigma – may have evolved in parallel. Similarly, the clade including samples resembling *E.perrieri*, *E.myriadenia* and *E.goudotiana* represents a wide range of Malagasy *Erica* variability, particularly of leaf shape and indument type, and these species are correspondingly dispersed across Perrier’s system. The rapid radiation of Malagasy *Erica* was apparently accompanied by morphological disparification, but whether this represents adaptive variation remains to be tested.

The additional ITS data added to the existing supermatrix of plastid and ITS/ETS data of Pirie et al. (2024) falls unambiguously within a monophyletic Malagasy/Mascarene clade but does not result in a well-supported internal topology. Undetermined collections that we tentatively grouped into morphospecies tended to show similar sequence variation (Fig. 2c), but such variation was low and the groupings unsupported. This is in contrast to within the *Erica* Cape clade, which represents an order of magnitude more species than the Malagasy clade but nevertheless shows enough variation in ITS to be able to test the relationships of individual specimens (Hoekstra et al., in press). The difference in sequence variation may be explained by a combination of younger age and/or faster diversification rates of Malagasy lineages. *Erica* is estimated to have arrived in Madagascar around 5–7 million years ago on the basis of relaxed clock molecular dating and a secondary Ericaceae calibration (Pirie et al. 2016). From these results, Pirie et al. (2016) estimated that the Malagasy clade may have one of the highest diversification rates in *Erica*. Given the dependence of these estimates on both absolute time calibration and relaxed clock assumptions, they should be revisited with further, phylogenomic data and ideally more calibration points. This would also allow reconstructing trait evolution within the Malagasy clade and testing e.g. for parallel evolution of insect pollination from a probably wind-pollinated ancestor.

## ﻿Distribution and habitats

We retrieved available georeferenced records for *Erica* and other Ericaceae from Madagascar via the Global Biodiversity Information Facility (GBIF.org, accessed 5 November 2024, https://doi.org/10.15468/dl.pmcvqr). They were filtered using R and CoordinateCleaner (Zizka et al. 2019) to remove potentially spurious occurrences, including records with a reported precision > 0.01° or 10 km and those with coordinates within 2 km of the country centroid, capital, or herbaria. This left 882 usable occurrences for *Erica*, 634 for *Agarista* and 872 for *Vaccinium*. Of the *Erica* occurrences, only 530 (60%) were identified to species. Twenty-five records were reported as (endemic) South African species, however in each case the species includes a synonym with an epithet matching a Malagasy *Philippia*. This is unlikely to be a coincidence. In these cases we assumed that the name originated by erroneously applying a *Philippia* epithet under *Erica*, and corrected the name following Dorr and Oliver (1999): “*Ericaaristata*” = *E.bojeri* Dorr & E.G.H.Oliv.; “*E.gracilis*” = *E.rakotozafyana* Dorr & E.G.H.Oliv.; “*E.hispida*” = *E.perhispida* Dorr & E.G.H.Oliv.; “*E.latifolia*” = *E.perrieri* Dorr & E.G.H.Oliv.; “*E.nudiflora*”, “*E.sparsa*”, and “*E.trichoclada*” all = *E baroniana* Dorr & E.G.H.Oliv. These cases highlight persisting confusion in the taxonomy of Malagasy *Erica*.

**Figure 3.**
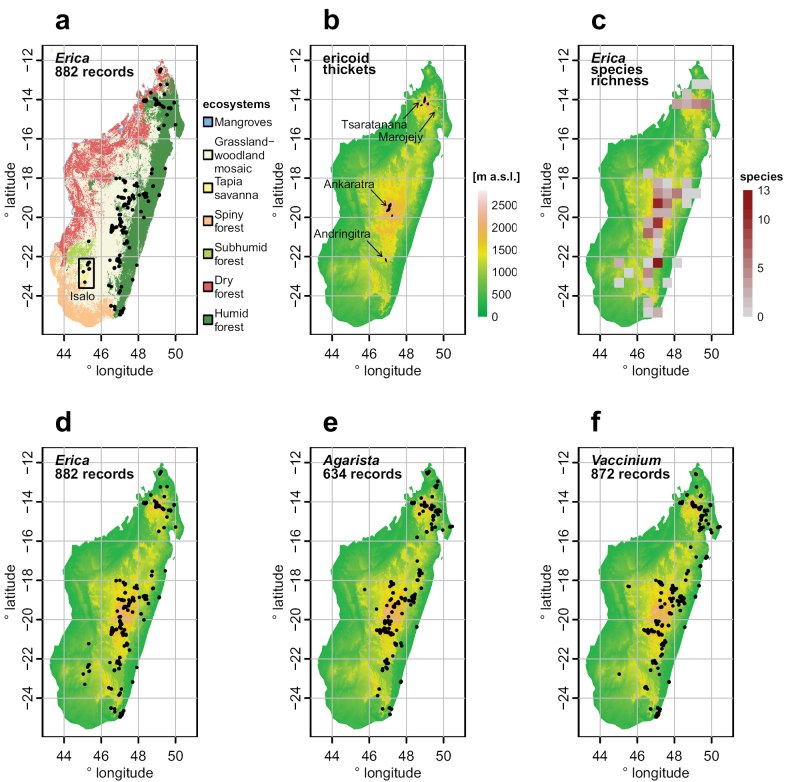
Distribution of *Erica* and other Ericaceae in Madagascar. Occurrence data were obtained from the Global Biodiversity Information Facility and submitted to multiple filtering steps (see text) **a***Erica* distribution plotted against the Madagascar ecosystem map of Antonelli et al. (2022). See also records mapped per species in Suppl. material 1**b** distribution of the “Madagascar ericoid thickets” ecoregion (Dinerstein et al. 2017) **c***Erica* species richness per 0.5 × 0.5 ° grid cell, summarised from occurrence records identified to species level **d** distribution of *Erica* in Madagascar compared to **e***Agarista* and **f***Vaccinium*, the other two genera of Ericaceae occurring in Madagascar.

Plotting *Erica* occurrences against Madagascar’s ecosystems (Fig. 3a), following the map of Antonelli et al. (2022), shows that *Erica* is found in all major ecosystem types except the western dry forests and the southwestern spiny forest (and the coastal mangroves). This includes the grassland–woodland mosaic of the central highlands, humid forest, and tapia savanna. Two specimens only identified to genus suggest *Erica* may also occur in the Makay massif in southwestern Madagascar, at the northern edge of the “subhumid forests” area. The “ericoid thickets” (Crowley 2004), described in some vegetation classifications and defined by *Erica* and similar “ericoid” shrubs, are confined to the four highest mountain massifs: Tsaratanana, Marojejey, Ankaratra and Andringitra (Fig. 3b). According to other authors, vegetation of this type also occurs further south, on the summit of Andohahela (Goodman et al. 2018c). The ericoid thickets cover only c. 1,300 km^2^ in total (Crowley 2004). The high mountains are also where the species richness of *Erica* is highest, peaking in the Ankaratra and Andringitra ranges, each with 13 species recorded in one 0.5 × 0.5° grid cell (Fig. 3c). Six species appear to be endemic to Andringitra alone (Appendix 1). Perrier de la Bâthie (1927b) named *Philippiafloribunda* Benth. (= *Ericabaroniana* Dorr & E.G.H. Oliv.) as the only species occurring at sea level in Madagascar, but *E.goudotiana* and *E.leucoclada* have also been recorded from the Eastern coast (Appendix 1, Suppl. material 1). The other two Ericaceae genera *Agarista* and *Vaccinium* have distribution patterns broadly similar to *Erica* in Madagascar (Fig. 3d–f); they have additionally been recorded from isolated humid forest areas of the western Bongolava region, which suggests *Erica* might also occur there.

**Figure 4.**
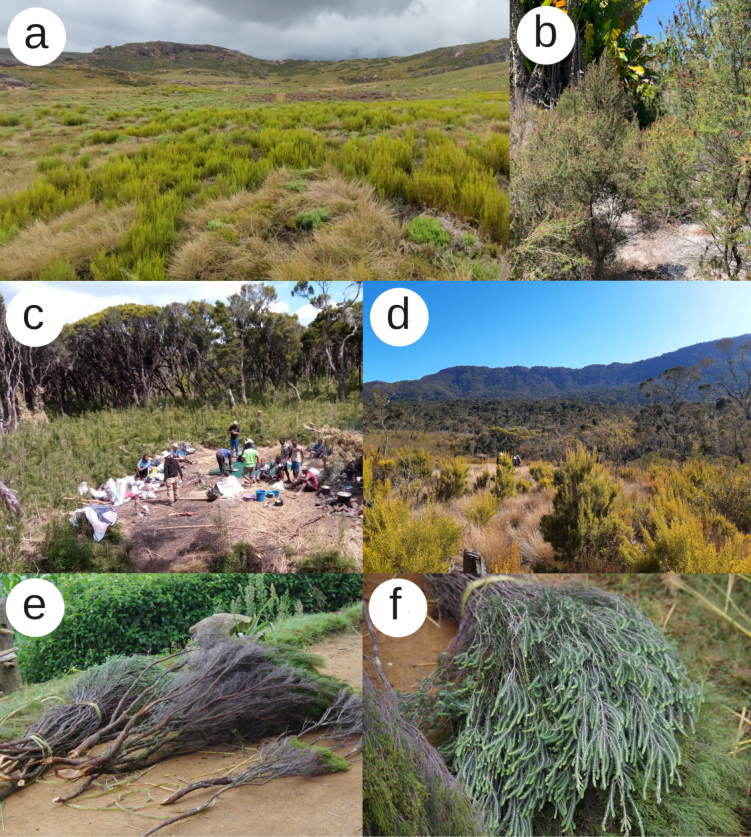
Habitats and uses of *Erica* in Madagascar **a***Erica* sp. in a mosaic with grassland, Andringitra National Park, Southeast, c. 2,000 m elevation. Lorna MacKinnon, Diana Rabeharison, Nantenaina Rakotomalala, and Fenitra Randrianarimanana 2022 **b***Erica* sp. on coastal sand, Manombo Special Reserve, Southeast. Nina Lester Finley 2023 (CC BY 4.0), https://www.inaturalist.org/observations/186728736**c** tree heathers, probably corresponding to Philippiacauliflorasubsp.gigas H.Perrier (now included in *Ericagoudotiana* (Klotzsch) Dorr & E.G.H.Oliv), at campsite Beanjavidy (“the big heathers”), Tsaratanana Reserve, North, c. 2,300 m elevation. Andry Rakotoarisoa 2022 **d***Erica* sp. colonising an opening dominated by the flammable grass Aristidacf.rufescens in Tsaratanana Reserve, at c. 2,000 m elevation. Jan Hackel 2022 **e–f** cut bundles of two unidentified *Erica* species near Anfanifotsy, just outside Andringitra National Park, c. 1,500 m elevation. Vincent Porcher 2020 (CC BY), https://www.inaturalist.org/observations/95638300.

*Erica* species in Madagascar are open-habitat shrubs or form their own canopy (Perrier de la Bâthie 1927b; Silander Jr et al. 2024). Based on the literature, there appear to be three main habitat types (see Fig. 4 for examples):

1. Ericoid thicket. This high-altitude vegetation was singled out as a distinct type under different names in treatments of the Malagasy flora (listed in Gautier et al. 2018), including the global ecoregions of Dinerstein et al. (2017). A 2004 ecoregion assessment classified its biodiversity as “globally endangered” and assigned it a conservation status of “Endangered” (Crowley 2004). It was included in the “humid forests” by Moat and Smith (2007) and Antonelli et al. (2022). In the ericoid thicket, *Erica* is associated with other shrubs of similar, “ericoid” appearance, especially *Helichrysum* (Asteraceae) and *Vaccinium* (Koechlin 1972). Perrier de la Bâthie (1927b) assumed this to be the original habitat of most *Erica* species in Madagascar, before it was supposedly destroyed by fire and *Erica* survived only in secondary vegetation. He also noted that *Erica* extends into the humid forest found in the zone below the ericoid belt, so the exact boundary of the ericoid thicket is somewhat fuzzy. Among the high-altitude species, Perrier de la Bâthie (1927b) cited three as specifically associated with the edges of marshes and bogs: *Philippiamadagascariensis* and *Ph.pilosa* (both now synonyms of *Ericamadagascariensis*), and *Ph.humbertii* (= *Ericahumbertii*). Dorr and Oliver (1999) described *Ericabosseri*, confined to marshes in Ankaratra.
2. Grassland–shrubland mosaic. This mixed vegetation type, difficult to classify (Antonelli et al. 2022), is found at lower altitudes, especially in the central highlands. The dominance of *Erica* shrubland versus grassland is probably dynamic and dependent on the fire regime (see below). In Ambohitantely Special Reserve in the central highlands, *Erica* forms part of the ecotone between grassland and forest (Ratsirarson et al. 2003). Perrier de la Bâthie (1927b, 1927a) interpreted most of the *Erica* shrubland outside the high-altitude areas as secondary, anthropogenic vegetation resulting from forest destruction through fire (“ *savoka à Philippia*”; *savoka* meaning fallow vegetation in Malagasy). The precise roles of fire regimes and land use in shaping the open vegetation of the central highlands are still debated (Joseph et al. 2021; Rakotoarivelo et al. 2024; Silander et al. 2024). In humid, high-altitude areas of Andringitra, the shrub-like, flammable grass *Panicumcupressifolium* appears to functionally mimic *Erica* (Lehmann et al. 2022). In eastern Madagascar, *Erica* is found in coastal habitat mosaics with grassland around littoral forest fragments. These open patches were also often interpreted as secondary vegetation (e.g. in Manombo Special Reserve, Goodman et al. 2018b). However, a palaeoecological study of littoral forest fragments around Mandena suggested that “ericoid grassland” with *Erica* and *Myrica* (Myricaceae) had already been part of a dynamic landscape before the arrival of humans (Virah-Sawmy et al. 2009a). A shift from forest to ericoid grassland around 1.400 cal. yr BP was attributed to the effects of sea level rise and drought rather than human action (Virah-Sawmy et al. 2009b).
3. Tapia savanna. This type of open-canopy vegetation, dominated by the tapia tree (*Uapacabojeri*, Phyllanthaceae), is found in several disjunct areas of the central highlands, with its largest extent in Isalo (Fig. 3a). It is adapted to fire and distinct from forest but also from the otherwise treeless grasslands of the central highlands (Kull 2002; Solofondranohatra et al. 2018). *Erica* forms part of the tapia understory alongside *Vaccinium* and other shrubs and smaller trees (Koechlin 1972; Gautier et al. 2018). *Ericadanguyana* (H.Perrier) Dorr & E.G.H.Oliv. and *E.jumellei* (H.Perrier) Dorr & E.G.H.Oliv. appear to be specifically associated with tapia savanna in the centre around Antsirabe and Arivonimamo, respectively; *Ericaibityensis* (H.Perrier) Dorr & E.G.H.Oliv., *E.lecomtei* (H.Perrier) Dorr & E.G.H.Oliv. and *E.isaloensis* may also be tapia-associated, according to their distributions on Mt. Ibity in central Madagascar (the former two) and the Isalo sandstone massif in the southwest; *Ericacryptoclada* (specifically the “var. *hybrida*” H.Perrier) was also found under tapia in Isalo, in addition to high altitude ericoid thicket (Perrier de la Bâthie 1927b; Dorr and Oliver 1999). Tapia savanna is actively managed by the local population: while the cutting of tapia trees is prohibited, *Erica* and other shrubs are extracted as fuelwood and for other uses (Kull 2002).

## ﻿Ecology and interactions

Malagasy *Erica* species appear to be adapted to fire. Perrier de la Bâthie (1921, 1927a, 1927b) viewed most open vegetation on Madagascar, including ericoid shrubland, as the result of degradation through fire set by humans. Today, it is generally accepted that fire was already part of Madagascar’s landscape well before human arrival (Kull and Lehmann 2022), and modern fire regimes are similar to those of other tropical regions (Phelps et al. 2022). At Lake Dangovavy, in the central highlands, ericoid shrubland was abundant during the Mid-Holocene, despite the occurrence of fires (Razafimanantsoa et al. 2025). *Erica* appear to be favoured by an infrequent fire regime: a study at Ambohitantely found *Erica* and Asteraceae shrubs appearing on sites that had not burnt for seven years (Randriatsara 2014). In Andringitra National Park, there has been a programme using fire to control *Erica* spread into open grassland for conservation purposes (Rasolonandrasana and Grenfell 2003), but a certain frequency of fire appears to favour *Erica* (Goodman et al. 2018a). At higher fire frequencies, grasses will dominate; at lower frequencies, trees may colonise and outcompete shrubs like *Erica* (Silander et al. 2024). It is likely that *Erica* in the central highlands historically formed part of a spatially shifting, decadal to centennial vegetation dynamic, as was shown in the ericoid belt of the Bale mountains of Ethiopia, where the palaeorecord evidenced a positive feedback between *Erica* abundance and fire (Gil-Romera et al. 2019). A study from southwest Madagascar suggested similar, spatially heterogeneous dynamics between littoral forest and *Erica*/*Myrica* grassland (Virah-Sawmy et al. 2009a). Today, fire regimes in the central highlands are almost exclusively determined by humans; fire is used, among other purposes, to control encroachment of *Erica* and other shrubs into grassland (Kull and Lehmann 2022).

We currently do not know to what degree Malagasy *Erica* species differ in fire adaptations. The high-altitude species of the ericoid thicket also appear to be very flammable, but it is unclear whether this represents an adaptation or a vulnerability. Some of the high-altitude species resprout quickly after fire (Perrier de la Bâthie 1927a). Natural, lightning-induced fires probably occur in the ericoid thicket (Crowley 2004; Gautier et al. 2018) – a possibility ruled out by Perrier de la Bâthie (1927a) – but at much lower frequencies than in the highlands or coastal areas. Perrier himself acknowledged the role of *Erica* as pioneer shrubs, quickly resprouting or colonising after fire and acting as nurseries for other plants (Perrier de la Bâthie 1927b). *Erica* species of the high mountains of East Africa were similarly found to be adapted to low-frequency fires (Fetene et al. 2006; Hemp 2006; Wesche 2006; Gil-Romera et al. 2019). Both ‘resprouter’ and ‘seeder’ fire strategies seem to occur among the Malagasy *Erica* (M.P., pers. obs.); observations from Cape *Erica* species (Ojeda 1998) suggest that the higher-altitude, more humid conditions would favour seeders, while the more pronounced drought season at lower elevation would select for resprouters. Surveys of *Erica* root systems could show whether there are species with lignotubers (Verdaguer and Ojeda 2002), and the impact of fire on seed germination could be compared among species. Growth form, leaf shape and leaf disposition probably also impact fuel properties (Keeley et al. 2011; Grootemaat et al. 2017) and should be examined for Malagasy *Erica*. This would help understand e.g. to what degree agricultural fires spreading from lower altitudes harm the ericoid thicket, and the conditions under which *Erica* stands fuel fires that can damage adjacent remnants of forest.

There has been little further research on the ecology of Malagasy *Erica*. Most species are found on the volcanic and metamorphic substrate of the high mountains and the lateritic soils of the central highlands. Some appear to be specific to quartzite plateaus or the southwestern Isalo sandstone range, and a single species, *Ericabarnettiana* Dorr & E.G.H.Oliv., was described from calcareous substrate, specifically, cipollino marble west of Ambositra (Perrier de la Bâthie 1927b). An endemic genus of springtails, *Anjavidiella*, is confined to litter under *Erica* on the high mountains; four species have been described from Ankaratra and Andringitra, but it has been suggested the genus may have radiated “as explosively as *Erica*” on the island (Betsch 2003). Ericoid mycorrhiza has not been studied in *Erica* or other Ericaceae in Madagascar. We also have not found any study of pollination in Malagasy *Erica*, but one of us (M.P.) has observed mitrastylous species flowering in the field: in contrast to those with a discoid stigma, they did not appear to release pollen into the air on disturbance, suggesting insect rather than wind pollination. Potential pollinators of these species remain to be identified. No seed information is available for Malagasy *Erica*; Perrier de la Bâthie (1927b) suggested the seeds disperse easily and retain their viability for long periods in the soil. He also hypothesised that migratory birds may have carried *Erica* seeds between Africa, Madagascar and the Mascarenes and specifically cited overlap in the ranges of the common quail, *Coturnixcoturnix*, and *Erica* in the central highlands of Madagascar (Perrier de la Bâthie 1927b).

**Figure 5.**
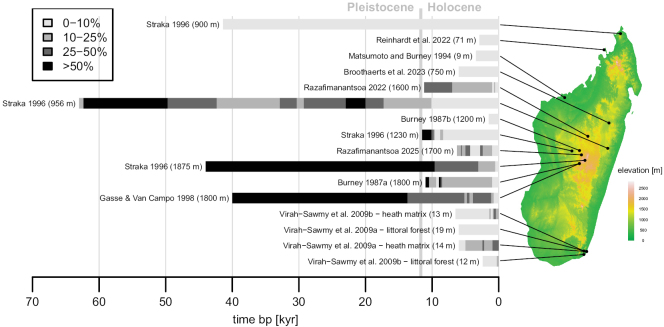
Abundance of Ericaceae pollen over time as proportion of overall pollen counts in sediment chronosequences in Madagascar. Data shown were summarised manually from 11 studies that recorded Ericaceae (see Suppl. material 3), varying in temporal extent and resolution. Only one study (Virah-Sawmy 2009b) identified *Erica* pollen to genus (*Vaccinium*, also identified, is not shown here). All other studies identified Ericaceae pollen only to family level. Data shown for Gasse & Van Campo (1998) are for “pollen group P1”, in which Ericaceae was the dominant taxon, and data for Virah-Sawmy (2009a) are for a pollen group grouping *Erica* with *Vaccinium*, Asteraceae, *Helichrysum* and graminoids. Data from Straka (1996) have only few calibration points and also include fern spores and aquatics in the total counts.

Ericaceae pollen has been recorded in Madagascar since the late Pleistocene (Fig. 5). We found 18 palynological studies of sediment chronosequences, mostly from lakes, of which 11 recorded Ericaceae pollen (Suppl. material 3). Only one study identified *Erica* pollen to genus (Virah-Sawmy et al. 2009b). The oldest sequence containing Ericaceae extended to c. 63 kyr cal BP (Torotorofotsy wetland in the eastern part of Madagascar; Straka 1996). Three sequences from the central highlands extending into the last glacial period of the Pleistocene (>11.7 kyr cal BP) recorded high to dominant levels of Ericaceae; this was interpreted as ericoid thicket spreading into lower-elevation zones during cooler and drier periods (Gasse and Van Campo 1998; Straka 2001). High levels of Ericaceae were also recorded in the gradually warming early Holocene in the central highlands, and levels then decreased toward the present (Burney 1987b, 1987a; Straka 1996; Razafimanantsoa 2022; Razafimanantsoa et al. 2025). Burney (1987b) noted that central highland pollen was “probably most likely *Philippia*, by far the most common ericoid genus in Madagascar”. By contrast, in modern *Erica* grassland on the southeastern coast, Virah-Sawmy et al. (2009a, 2009b) found an increase of Ericaceae and associated taxa toward the present, especially around 950 BP, interpreted as a shift from forest and woodland to *Erica* grassland. Finally, sequences from the eastern Alaotra wetland system, eastern littoral forest fragments, and from the north and northwest, recorded only low levels of 10% or less throughout the sequences (Matsumoto and Burney 1994; Straka 1996; Virah-Sawmy et al. 2009b; Reinhardt et al. 2022; Broothaerts et al. 2023).

The overall picture of a high-mountain ericoid thicket being widespread in highland Madagascar during the last glacial period and then receding to the high mountains appears consistent with reconstructed palaeoclimate (Gasse and Van Campo 1998). However, the present distribution of *Erica*, and other Ericaceae, extends far beyond the ericoid thicket biome (Fig. 3). There are few published pollen spectra from modern vegetation in Madagascar that can be used as references for the interpretation of ancient pollen counts (Burney 1988; Straka 1991; Razafimanantsoa and Razanatsoa 2024). *Erica* can form dense thickets in the present-day central highlands (see Ecology section), which could also yield high local pollen counts. The studies of Virah-Sawmy et al. (2009b, 2009a), set near the south-eastern coast, also show that high levels of *Erica* may reflect an environment quite different from the montane thickets. The lack of resources for species identification, knowledge of the ecology of *Erica* and other Ericaceae, and clearly identified pollen reference material, also severely limits the interpretation of the palaeontological record. Ericaceae pollen, including from *Erica*, occurs mostly in tetrads, and the exine is characterised by the presence of numerous minute granules as secondary sculpture (Sarwar and Takahashi 2014). Straka (1991) noted for Madagascar that “it is impossible to distinguish *Philippia* [pollen] tetrads”, based on light microscopy. However, a recent scanning-electron microscope study of *Erica* pollen found differences in pollen aggregation, size, shape and exine ornamentation between species (Wrońska-Pilarek et al. 2018). Four Malagasy taxa included in this study all had pollen in tetrads but differed in other aspects of pollen morphology. This suggests that the collection and characterisation of pollen reference material for the extant Malagasy *Erica* species may yield features diagnostic for species or at least species groups, which combined with better knowledge on the ecology, may improve the resolution of the palaeo-record.

## ﻿Uses and conservation

Only a few traditional uses have been documented for Malagasy *Erica* species. They are used mainly by Merina and Betsileo people from the central highlands, but also by Betsimisaraka people from the eastern region. The genus *Erica* is clearly delimited from *Agarista* and *Vaccinium* in different dialects of the Malagasy language, with local names reflecting ecological knowledge and uses. For *Agarista* and *Vaccinium* respectively, 15 and 17 local names have been reported, mostly corresponding to clearly identified species (Schatz 2005), but for *Erica* only five: *anjavidilahy*, *anjavidy*, *riadriatra*, *kisiasia* and *anjavidilahimadinika* – the most commonly used being *anjavidy*, an ethnospecies encompassing several taxonomic species. The term *anjavidy* is formed from *anjaka*, “the action of tying into bundles” and *vidy*: ”price, value“, which refers to its main use as firewood (de Veyrières and de Méritens 1967; Boiteau et al. 1980). In the past, trade in heather as firewood was particularly important and offered high returns (Boiteau et al. 1980). This wood was mainly used by affluent families and was highly appreciated for its fragrance when burned. The term *riadriatra*, mainly used by Betsileo, also refers to this use, being built on *riatra*: “which makes noise when set on fire”. By extension, *anjavidy* can also refer to species other than *Erica* that share similar characteristics (mainly flammability), such as *Hibbertiacoriacea* Baill. (Dilleniaceae) and *Myrothamnusmoschatus* (Baill.) Baill. ex Nied. (Myrothamnaceae) (Boiteau et al. 1980).

While the most widespread use of *Erica* in Madagascar is for fuel (Gade and Perkins-Belgram 1986), species of the genus probably have other uses, not all of which are documented. *Erica* branches are used as brooms (*kifafa-anjavidy*, “heather brooms”; Fig. 4e, f), but also as thatch for the roofs of Betsileo houses and as the preferred support for silkworm cocoons in traditional nurseries (Perrier de la Bâthie 1927b; Vernier 1964). In addition, some studies mention the use of *Erica* in traditional medicine: as cough treatment (Kull 2002), anti-fever decoction from leafy branches (Onjalalaina et al. 2021) or for treating wounds, according to Betsileo traditional practitioners (V.P., unpublished). Finally, heathers are used by farmers as indicator plants for exhausted soils. After the passage of fire on an agricultural or forest plot, heather forms a dense secondary vegetation known as *savok’anjavidy* (literally, “heather fallow”). Farmers avoid these areas for cultivation (Carrière et al. 2005).

**Figure 6.**
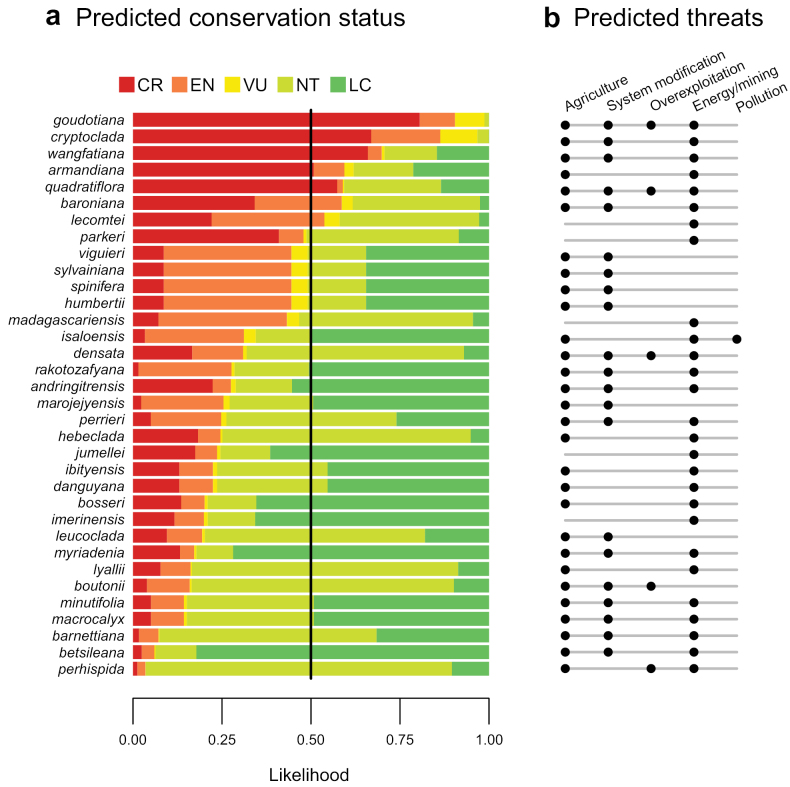
Predicted conservation status and threats for Malagasy *Erica* species, from Ralimanana et al. (2022). International Union for the Conservation of Nature categories for (a) conservation status and (b) threats were predicted for all unassessed plant species of Madagascar using a neural network model based on species occurrences and associated environmental predictors, with existing plant assessments as training set (Zizka et al. 2022). CR: critically endangered, EN: endangered, VU: vulnerable, NT: near threatened, LC: least concern. *Ericabojeri* was not assessed.

The conservation status of Malagasy *Erica* has not been formally assessed under the International Union for the Conservation of Nature (IUCN) red list criteria. However, at least one-fifth of the Malagasy species are likely to be threatened, according to a machine learning prediction for the whole of Madagascar’s flora, based on recorded occurrences and environmental predictors (Ralimanana et al. 2022); Fig. 6). Seven out of 34 assessed *Erica* species (20.5%) had a combined likelihood > 0.5 to be threatened (critically endangered, endangered or vulnerable; Fig. 6a); of these, five had a likelihood > 0.5 to be critically endangered: *Ericaarmandiana* Dorr & E.G.H.Oliv., *E.cryptoclada*, *E.goudotiana*, *E.quadratiflora* and *E.wangfatiana* Dorr & E.G.H.Oliv. Surprisingly, this includes two rather widespread species (*E.cryptoclada* and *E.goudotiana*). “Agriculture” was predicted as a likely threat for 29 of the Malagasy *Erica* species, which is in line with the results of Ralimanana et al. (2022) for the whole of the Malagasy flora, where Agriculture and overexploitation affected 90% of all plant species. The central highlands, corresponding to much of the *Erica* range, are the most densely populated area of Madagascar and used for field cultivation and cattle farming. “Energy/mining” was predicted as a threat for 27 species and appears likely, given the widespread use of *Erica* as firewood; a better understanding of the species collected will be important. Southern littoral habitats in particular have been cleared for mining purposes (Virah-Sawmy 2009). “System modification” was predicted for 21 species; this could correspond to changing fire regimes. Fire was cited as the most important threat for the high-altitude ericoid thicket (Crowley 2004), but many *Erica* species also seem to require a minimum fire regime (see Ecology section above). “Overexploitation” overlaps with use for “Energy” in the case of *Erica* and was only predicted for five species, and “Pollution” only for one species. More basic research on individual *Erica* species is needed to identify threats and enable formal conservation assessments.

## ﻿Conclusion and research priorities

1. Taxonomic revision. The synthesis of taxonomy and diagnostic traits that we have now incorporated into WFO and the *Erica* Identification Aid should help to some extent in identifying *Erica* species in Madagascar. Particularly for non-specialists, the combination of Latin and French descriptions presented in Perrier de la Bâthie (1927a) may not have been straightforward to interpret. However, this is unlikely to have been the main stumbling block leading to the high proportions of undetermined collections apparent today. Perrier’s work was based on material available around 100 years ago, and his approach in defining relatively narrow species concepts whilst describing numerous exceptions might reflect real, complex variation but does not necessarily help in putting names to specimens. A modern revision integrating molecular data to test species boundaries is sorely needed.
2. Phylogenomics. Phylogenetic marker sets employed so far are clearly not sufficient to resolve relationships within the Malagasy clade. More markers with greater numbers of variable characters, e.g. using targeted sequence capture (Musker et al. 2024), will be needed both to improve resolution and to test hypotheses of hybridisation, biogeography and trait evolution in this rapid radiation. Uncertainty remains as to the positions of 22 Malagasy species which are either yet to be sequenced or cannot yet be unambiguously attributed to undetermined collections (Appendix 1).
3. Ecology. Very little ecological information can currently be attributed to species, and Malagasy *Erica* are commonly treated as a single functional group. Yet the broad distribution in Madagascar and the radiation into at least 35 species suggest significant variation. The existence of both wind- and insect-pollinated species should be verified in the field. Better understanding of the variation in fire and disturbance ecology between species would directly inform land management and conservation. Improving resolution and functional interpretation of the pollen record would contribute to a better understanding of the central highlands’ debated vegetation history. Linking field studies and the collection of trait data to herbarium voucher specimens will be essential for tracing observed variation through an evolving taxonomy.
4. Conservation. The lack of taxonomic revision and resources currently hinder formal conservation assessments. The preliminary estimate that at least one-fifth of the Malagasy *Erica* species are threatened appears conservative in light of the threats facing Madagascar’s biota in general (Ralimanana et al. 2022). Missing taxonomic and ecological information also prevents effective in situ and ex situ conservation. While species of the high-altitude thickets may mostly be covered by existing protected areas, the status of central highland and coastal species is unclear. Only two ex situ collections of Malagasy *Erica* exist in the Millennium Seed Bank as of November 2024, both with unclear species identities. Finally, recording traditional uses, which likely include more than the ones we reported, will both aid in designing effective conservation strategies and help preserve traditional knowledge.

## ﻿Additional information

### Author ORCIDs

Jan Hackel https://orcid.org/0000-0002-9657-5372

Andriantsilavo H. I. Razafimanantsoa https://orcid.org/0000-0002-9933-6991

Vincent Porcher https://orcid.org/0000-0003-2879-6728

Michael D. Pirie https://orcid.org/0000-0003-0403-4470

### Data availability

All of the data that support the findings of this study are available in the main text or Supplementary Information.

## ﻿Appendix 1

**Table A1.** The 35 species of *Erica* L. currently accepted in Madagascar (Antonelli et al. 2022; Elliott et al. 2024; World Flora Online 2024), with World Flora Online (WFO) and Global Biodiversity Information Facility (GBIF) identifiers and notes on distribution and ecology (unless otherwise specified, following Perrier^1^) and diagnostic traits (mostly summarised from Perrier^1^). We include Perrier’s species numbering system (#)^1^ with subsequently described species incorporated according to documented similarity, and Perrier’s species groups based on *Philippia* epithets^3^. Sources: ^1^Perrier de la Bâthie (1927), ^2^Dorr and Oliver (1999), ^3^Perrier de la Bâthie (1930), ^4^GBIF occurrence data (https://doi.org/10.15468/dl.pmcvqr) – see also species occurrence maps in Suppl. material 1.

Species	Distribution	DNA data	Diagnostic description	Perrier’s *Philippia* group (#)	Notes
*Ericaandringitrensis* (H. Perrier) Dorr & E.G.H.Oliv. https://list.worldfloraonline.org/wfo-0000671368; https://www.gbif.org/species/5683023	Andringitra¹	N	Branched shrub, 1-2 m; leaves in whorls of 4; flowers solitary, arranged in leafy spike-like inflorescences along branches; stamens free, discoid stigma with inconspicuous lobes.	*trichoclada* but also similar to *cauliflora* (9)	
*Ericaarmandiana* Dorr & E.G.H.Oliv. https://list.worldfloraonline.org/wfo-0000671417; https://www.gbif.org/species/5682917	Central highlands²	N	Plants more or less glandular, leaves in whorls of 4; flowers in terminal clusters; pedicel 2 mm long, surrounded by slightly differentiated leaves; large, bristly, glandular sepal without a dorsal slit; filaments fused only at the base; stigma lobes not extending beyond disc.	*trichoclada* (18)	Similar to *E.hebeclada* and may fall within the variation in that species.
*Ericabarnettiana* Dorr & E.G.H.Oliv. https://list.worldfloraonline.org/wfo-0000671480; https://www.gbif.org/species/5682775	Central highlands (Ivato basin west of Ambositra)¹	N	Narrowly branched shrub with white-pubescent branches; leaves opposite, superimposed in 4 rows completely hiding the stem; stamens connate; stigma lobes not extending beyond disc.	- (8)	The only Malagasy species growing on limestone.¹
*Ericabaroniana* Dorr & E.G.H.Oliv. https://list.worldfloraonline.org/wfo-0000671481; https://www.gbif.org/species/5682773	Central highlands and eastern coast¹	N	Erect shrub (“*Ericascoparia*-like habit”) with spindly greyish pubescent branches; leaves in whorls of 3 (2 on young branches), 2-4 mm long, spreading or ascending but not adnate to stem; flowers in terminal clusters of 3-7, not subtended by differentiated leaves; corolla 1.3 mm long, valvate; stigma lobes not extending beyond disc.	*floribunda* (34)	Common, widespread and very variable.
*Ericabetsileana* (H. Perrier) Dorr & E.G.H.Oliv. https://list.worldfloraonline.org/wfo-0000671503; https://www.gbif.org/species/5682711	Central highlands: quartzite mountains between Ivato and Mania basins¹	N	Lax, green shrub; leaves distant, spreading/recurved; short glandular bristles; 8 stamens adnate at base; 4 long stigmatic lobes and reflexed tubular disc.	*pilosa* but also similar to *trichoclada* (5)	Similar to *E.parkeri* and *E.humbertii*, differing in habit (shrubby in *E.parkeri*) and indument (long non-glandular bristles in *E.humbertii*)
*Ericabojeri* Dorr & E.G.H.Oliv. https://list.worldfloraonline.org/wfo-0000671524; https://www.gbif.org/species/5682663	Central highlands (Imerina)²	Y	Stem hairy, brownish; internodes longer than the leaves; leaves in whorls of 4; petiole 0.25 mm, blade 1.5-2 mm long, dorsal and ventral surfaces domed, with a very narrow dorsal slit, covered with small, fairly sparse brownish hairs, with a long-stalked (up to 2/3 as long as the leaf) gland at the apex; flowers in terminal clusters (3-5), pedicel 2 to 3 mm long, not surrounded by differentiated leaves; calyx with obtuse, pubescent lobes bearing a few glands, the largest without a dorsal slit, reaching the middle of the corolla; 7-8 stamens with filaments free to the base; style strongly bristly towards the (swollen) base, stigma lobes not extending beyond disc.	*trichoclada* (21)	Similar to *E.hebeclada*, maybe just a variant.¹
*Ericabosseri* Dorr https://list.worldfloraonline.org/wfo-0000671534; https://www.gbif.org/species/5682642	Ankaratra²	N	Low, erect or decumbent shrub 0.3 m tall with reddish pubescent branches; leaves with glandular and simple hairs, terminating in a very stout, 0.8-1 mm long, gland-tipped hair that forms an acute angle with the plane of the leaf blade; calyx lobes broadly obovate or narrowly oblanceolate to oblanceolate with glandular pubescence; narrow corolla mouth, anthers included within the corolla, style hairy with 4 long stigmatic lobes and reflexed tubular disc.	*pilosa* (1b)	Similar to *E.madagascariensis* from which it differs chiefly in the hairs of the leaves, in the shape and size of the sepals, and especially in the shape of the corolla.²
*Ericaboutonii* Dorr & E.G.H.Oliv. https://list.worldfloraonline.org/wfo-0000671540; https://www.gbif.org/species/5682634	Central highlands (Imerina)¹	Y	Shrub with white-pubescent branches but without glandular hairs, leaves in whorls of 4, overlapping and hiding the stem; flowers in terminal clusters surrounded by slightly differentiated leaves, stigma discoid with slightly projecting lobes.	*ciliata* (11)	Similar to *E.leucoclada* which has stamens fused on lower half only and broader stigmas. The var.cinerea¹ has greyish appearance due to denser indument.
*Ericacryptoclada* (Baker) Dorr & E.G.H.Oliv. https://list.worldfloraonline.org/wfo-0000671793; https://www.gbif.org/species/5682053	Central highlands, (Tsarantanana and Marojejy to Isalo)¹ – records also from Andohahela^4^	Y	Shrub with white pubescent branches; leaves in whorls of 3, < 6 mm long, very appressed on the stem, closely imbricate, with sessile black corpuscles on the margins; flowers in terminal clusters of 3-7, not subtended by differentiated leaves, corolla <2 mm long with overlapping lobes with toothed margins, stigma lobes visible.	*gracilis* (30)	Widespread and variable; “var.hybrida” H.Perrier (considered synonymous²) found under tapia in Isalo.
*Ericadanguyana* (H. Perrier) Dorr & E.G.H.Oliv. https://list.worldfloraonline.org/wfo-0000671839; https://www.gbif.org/species/5681963	Central highlands (Antsirabe)¹,³	N	Shrub of 1 to 2 m., hairy but without glands, leaves ≥4x as long as wide, in whorls of 4, not hiding stem; flowers in terminal clusters, 8 connate stamens, discoid stigma with inconspicuous lobes.	*ciliata* (10)	Similar to *E.boutonii* but differing in longer leaves and pedicels and in the larger, thick sepals that extend beyond the corolla; see also *E.quadratiflora*
*Ericadensata* Dorr & E.G.H.Oliv. https://list.worldfloraonline.org/wfo-0000671864; https://www.gbif.org/species/5681923	Disjunct populations at Manongarivo and Tsaratanana in the north; central highlandst¹	N	Shrub with tomentose branches; hairs non-glandular, branched; leaves in whorls of 3 or 4, internodes c. 1mm (shorter than diameter of stem), overlapping and hiding the stem; flowers in terminal clusters surrounded by slightly differentiated leaves, stigma lobes not extending beyond disc.	*ciliata* (16)	Differs from *E.boutonii* by the much thicker, larger calyx with keeled sepals, at least the largest of which has a dorsal slit. Distinct local forms (specimens from the north reaching 3-4 m)¹.
*Ericagoudotiana* (Klotzsch) Dorr & E.G.H.Oliv. https://list.worldfloraonline.org/wfo-0000672181; https://www.gbif.org/species/8632971	Tsaratanana, Central Highlands, Itremo, East coast¹	N	Shrub 1-2 m tall (but see wider variation), branches with white indument, leaves in whorls of 3, largely glabrous, without spines at apices; flowers solitary, subtended by 3-4 narrow, yellowish leaves, largest sepal as long as petals, the rest reaching bases of petal lobes, all glabrous but ciliate at margins; stigma lobes inconspicuous.	*floribunda* and *cauliflora* (*Ph.cauliflora* now considered a synonym) (24)	Perrier’s description matches the common form only: he described subspecific taxa (such as ‘*Philippiacaulifloragigas*’, reaching 5-6 m in height) and reported ‘innumerable’ deviating forms.¹ Species complex.
*Ericahebeclada* Dorr & E.G.H.Oliv. https://list.worldfloraonline.org/wfo-0000672221; https://www.gbif.org/species/5684867	Central highlands, Ankaratra, Andringitra¹ – records also from Isalo^4^	Y	Shrub with upright habit; stem whitish pubescent; abundance of glands on the stems, leaves, pedicels and calyces; leaves in whorls of 4 often ascending and elongated; petioles 0.6-1 mm long, blade 2 to 3 mm, domed above, with a clear dorsal slit; flowers in terminal clusters (3-6), pedicel 1.5 mm long, not surrounded by differentiated leaves; calyx bristly, glandular, largest sepal without a dorsal slit, not reaching the corolla sinuses; filaments entirely fused; style bristly at base, stigma lobes not extending beyond disc but clearly visible in profile on the fresh flower.	*trichoclada* (19)	Variable: two varieties differing in e.g. leaf shape and distribution of indument¹.
*Ericahumbertii* (H. Perrier) Dorr & E.G.H.Oliv. https://list.worldfloraonline.org/wfo-0000672270; https://www.gbif.org/species/5684738	Andringitra¹	Y	Low sprawling green plant, leaves distant, spreading/recurved, very long non-glandular bristles on stems and leaf tips, 3-4(-6) stamens, 4 long stigmatic lobes and reflexed tubular ‘disk.	*pilosa* (3)	
*Ericaibityensis* (H. Perrier) Dorr & E.G.H.Oliv. https://list.worldfloraonline.org/wfo-0000672285; https://www.gbif.org/species/5684706	Central highlands (Ibity)¹	N	Shrub up to 1.2 m, stiff grey pubescent branches, without glandular hairs, leaves in whorls of 4, overlapping and hiding the stem; flowers in terminal clusters surrounded by wider, thinner, sessile leaves, stigma lobes inconspicuous.	*ciliata* (14)	
*Ericaimerinensis* (H. Perrier) Dorr & E.G.H.Oliv. https://list.worldfloraonline.org/wfo-0000672293; https://www.gbif.org/species/5684685	Central highlands: south of Tsinjoarivo¹	N	Rigid shrubs with white pubescent branches; leaves in whorls of 3, <6 mm long, narrowly imbricate, loosely erect against the stem and hiding it, glabrous but with very short, stalked glands when young; flowers in terminal clusters of 3-7, not subtended by differentiated leaves, corolla <2 mm long with overlapping lobes, stigma lobes inconspicuous.	- (29)	
*Ericaisaloensis* (H. Perrier) Dorr & E.G.H.Oliv. https://list.worldfloraonline.org/wfo-0000672347; https://www.gbif.org/species/5684596	Isalo¹,³	Y	Dwarf plant; leaves in whorls of 4, upright hiding the stem, with domed upper surfaces and narrow slit; corolla glabrous, 8 free stamens, stigma with prominent lobes extending beyond disc.	*pilosa* but also similar to *trichoclada* (7)	
*Ericajumellei* (H. Perrier) Dorr & E.G.H.Oliv. https://list.worldfloraonline.org/wfo-0000672364; https://www.gbif.org/species/5684573	Central highlands (Arivonimamo)¹	N	Shrub with slender branches (1.5-2.5 mm diam.), without glandular hairs, leaves in whorls of 3 (2, 4), overlapping and hiding the stem; flowers in terminal clusters surrounded by slightly differentiated leaves, stigma discoid with inconspicuous lobes.	*ciliata* (12)	Similar to *E.densata* and *E.boutonii* but leaves and flowers are smaller; sepals shorter and not split at the back (cf. *E.densata*); and with a large carinate sepal (cf. *E.boutonii*)¹.
*Ericalecomtei* (H. Perrier) Dorr & E.G.H.Oliv. https://list.worldfloraonline.org/wfo-0000672443; https://www.gbif.org/species/7328672	- subsp.lecomtei: central highlands (Ibity, Itremo)²; - subsp.ravinakely Dorr: Isalo²	N	Shrub 1 to 4 m tall; leaves in whorls of 3, 8-10 mm long, erect hiding the stem, narrow-lanceolate-linear with scaly, denticulate margins, dorsal slit narrow, leaving neither the midrib nor the underside of the leaf blade visible; flowers in terminal clusters of 3-5, not subtended by differentiated leaves; corolla 5 mm long with 2 mm lobes, stigma lobes not extending beyond disc.	*floribunda* but also similar to *ciliata* (28)	Similar to *E.viguieri*.
*Ericaleucoclada* (Baker) Dorr & E.G.H.Oliv. https://list.worldfloraonline.org/wfo-0000672464; https://www.gbif.org/species/5684343	Central highlands (North Antsihanaka) – records also from Mandena (east coast)^4^	N	Shrub with white-pubescent branches but without glandular hairs, leaves in whorls of 4 (3,2), overlapping and hiding the stem; flowers in terminal clusters surrounded by slightly differentiated leaves, filaments fused along lower half, stigma discoid, broad (1.25 mm), with slightly protruding lobes.	*ciliata* (13)	
*Ericalyallii* Dorr & E.G.H.Oliv. https://list.worldfloraonline.org/wfo-0000672520; https://www.gbif.org/species/5684247	Central highlands (Ankaratra, Ibity)¹ – records also from Kalambatritra (south)^4^	N	See *E.baroniana*.	*floribunda* (34b)	Considered doubtful¹; perhaps a form of *E.baroniana* or similar. *Philippiafloribundaglandulosa* H. Perrier synonymous².
*Ericamacrocalyx* (Baker) Dorr & E.G.H.Oliv. https://list.worldfloraonline.org/wfo-0000672539; https://www.gbif.org/species/5684207	Central highlands¹	N	Shrub with bristly branches without glandular hairs, leaves in whorls of 4, overlapping and hiding the stem; flowers in terminal clusters surrounded by slightly differentiated leaves, filaments fused, stigma not exserted, discoid, 1 mm diam., with raised margins hiding the lobes.	*ciliata* (15)	
*Ericamadagascariensis* Dorr & E.G.H.Oliv. https://list.worldfloraonline.org/wfo-0000672547; https://www.gbif.org/species/5684195	Central highlands, Ankaratra²	Y	Low growing, very hairy greyish shrub, erect leaves hiding stems; sepals narrow, long ciliate; style hairy with 4 long stigmatic lobes and reflexed tubular disc.	*pilosa* (1/2)	Similar to *E.bosseri*.
*Ericamarojejyensis* Dorr https://list.worldfloraonline.org/wfo-0000672576; https://www.gbif.org/species/5684138	Marojejy²	N	Distinguished from *E.lecomtei* by its spreading (versus imbricate and rigidly ascending) leaves that are pubescent (versus glabrous) adaxially and by its calyx in which the longest calyx lobe slightly exceeds the corolla in length. The longest calyx lobe in *E.lecomtei* is shorter than the corolla in length. Distinguished from *E.viguieri* by its shorter (2.5-5.2 versus 6.5-12 mm long) and narrower (1.5-2 versus 2-2.8 mm wide) leaves that have a closed (versus open) sulcus. Additionally, the inflorescences of E. *marojejyensis* have many fewer (3 versus 9) flowers than those of *E.viguieri*.	- (28b)	Similar to *E.lecomtei* and *E.viguieri*².
*Ericaminutifolia* (Baker) Dorr & E.G.H.Oliv. https://list.worldfloraonline.org/wfo-0000672623; https://www.gbif.org/species/5684041	Central highlands²	N	Stems greyish pubescent with short stalked glands; leaves usually 4; petiole 0.25-0.5 mm; blade 1-1.5, with small protuberances on the margins; flowers in terminal clusters, pedicel 1.5-3 mm long, bristly glandular, not surrounded by differentiated leaves, the large sepal without a dorsal slit slightly exceeding the middle of the corolla; 8 stamens with filaments united at the base; style long (2.5 mm) with a few bristles towards the base, stigma lobes not extending beyond disc.	*trichoclada* (22/23)	Similar to *E.bojeri*; includes Perrier’s concept of *Ph.oophylla* which represents variation with larger leaves and longer pedicels.
*Ericamyriadenia* (Baker) Dorr & E.G.H.Oliv. https://list.worldfloraonline.org/wfo-0000672681; https://www.gbif.org/species/5683931	Central highlands¹	Y	Plants more or less glandular, leaves in whorls of 4, flowers in terminal clusters, sessile, surrounded by slightly differentiated leaves, with large, bristly, glandular sepal without a dorsal slit, filaments entirely fused, stigma lobes not extending beyond disc.	*trichoclada* but also similar to *ciliata* (17)	Widespread and variable¹.
*Ericaparkeri* (Baker) Dorr & E.G.H.Oliv. https://list.worldfloraonline.org/wfo-0000672822; https://www.gbif.org/species/5683749	Central highlands¹	Y	Shrubby green plant, leaves distant, spreading/recurved, short glandular bristles, 8 adnate stamens, 4 long stigmatic lobes and reflexed tubular disc.	*pilosa* (4)	Very variable; differs from *E.humbertii* by its glandular bristles, and adnate (as opposed to free) stamens¹.
*Ericaperhispida* Dorr & E.G.H.Oliv. https://list.worldfloraonline.org/wfo-0000672870; https://www.gbif.org/species/5683659	Central highlands, Tsaratanana¹	N	Spindly, recumbent plant, all parts with glandular hairs; leaves in whorls of 4, petiole 0.5-0.75 mm, blade variable, 1-2 mm, oval or ovate-lanceolate on the same branch, backs somewhat open; flowers in terminal clusters (3-5), not surrounded by differentiated leaves, 1-1.5 mm long, largest sepal without a dorsal slit; 8 stamens fused partly or almost to the apex, style bristly at base, stigma lobes prominent, extending beyond disc (or not).	*pilosa* (*Ph.hispida*) and *trichoclada* (*Ph.adenophylla* (6/20)	Status of local variant “*Philippiahispidaangustifolia*” H. Perrier from Tsaratanana unclear.² *Philippiaadenophylla* Baker (#20) considered synonymous²
*Ericaperrieri* Dorr & E.G.H.Oliv. https://list.worldfloraonline.org/wfo-0000672877; https://www.gbif.org/species/5683647	Andringitra, Ibity¹	Y	Branched shrub 1-4 m tall, leaves in whorls of 3, ovate, open-backed, >6 mm long, glabrous; flowers in terminal clusters of 3-7, not subtended by differentiated leaves; stigma lobes visible.	- (26)	
*Ericaquadratiflora* (H. Perrier) Dorr & E.G.H.Oliv. https://list.worldfloraonline.org/wfo-0000673059; https://www.gbif.org/species/5683223	Andohahela²,³ – widely disjunct records also from the northern Sambava region^4^		Entirely glabrous shrub with white branches, leaves in whorls of 4, 4-7 mm long, lanceolate, backs entirely closed; flowers in terminal clusters (6-12), tinted red (as are surrounding leaves), 5-6 mm long, largest sepal attenuated into a sharp point, with a long dorsal slit; 8 stamens united almost to the apex, style thick, 1 mm long with a 3 mm wide stigmatic disc with prominent lobes.	*ciliata* (11b)	Perrier (1930) compared this new species to *E.danguyana*, which differs in being hairy, and having smaller leaves and flowers
*Ericarakotozafyana* Dorr & E.G.H.Oliv. https://list.worldfloraonline.org/wfo-0000673076; https://www.gbif.org/species/5683190	Central highlands, Andringitra¹ – records also from the southeastern coast³	Y	Shrub up to 1 m tall, many slender branches with greyish indument; leaves in whorls of 3 (often 2 on young branches), < 6 mm long, narrowly imbricate, appressed, blackish; flowers in terminal clusters of 3-7, not subtended by differentiated leaves, corolla <2 mm long with overlapping lobes, stigma lobes not extending beyond disc.	*gracilis* (31)	
*Ericaspinifera* (H. Perrier) Dorr & E.G.H.Oliv. https://list.worldfloraonline.org/wfo-0000673295; https://www.gbif.org/species/5682712	Andringitra¹	Y	Shrub 1-6 m tall, branches with grey indument; leaves in whorls of 3, serrated at margins, with 1.5 mm or longer ‘spines’ at apices (formed from adnate hairs; lost in older leaves); flowers solitary, subtended by 3-4 narrow, yellowish leaves, larger calyx lobe almost free, the others fused in lower third; stigma lobes inconspicuous.	*ciliata* but also similar to *cauliflora* (25)	
*Ericasylvainiana* Dorr & E.G.H.Oliv. https://list.worldfloraonline.org/wfo-0000673362; https://www.gbif.org/species/5682545	Andringitra¹	N	Low growing, cypress-like habit with rigid, slender, white-pubescent branches, other parts appearing glabrous; leaves in whorls of 3, 1-3 mm long, dimorphic: much smaller on flowering stems, spreading or ascending but not adnate to stem; flowers in terminal clusters of 3-7, not subtended by differentiated leaves, corolla 1.25 mm long, valvate, stigma lobes not extending beyond disc.	*floribunda* (33)	
*Ericaviguieri* (H. Perrier) Dorr & E.G.H.Oliv. https://list.worldfloraonline.org/wfo-0000673574; https://www.gbif.org/species/5681980	Andringitra¹	N	Shrub 2-4 m tall, thick stem with very visible, closely spaced leaf scars; leaves in whorls of 3, 8-10 mm long, narrow-lanceolate-linear with scaly, denticulate margins, a dorsal slit revealing prominent midrib; flowers in large, nodding terminal clusters of 3-7, not subtended by differentiated leaves; corolla 4.5 mm long with 2 mm lobes, stigma lobes inconspicuous.	*floribunda* but also similar to *ciliata* (27)	Similar to *E.lecomtei*.
*Ericawangfatiana* Dorr & E.G.H.Oliv. https://list.worldfloraonline.org/wfo-0000673625; https://www.gbif.org/species/5681890	Andringitra¹	N	Rigid shrub with glabrous branches; leaves in whorls of 3, 2-6 mm long, half as short on small branches than on the others spreading or ascending but not adnate to stem; flowers in terminal clusters of 3-7, not subtended by differentiated leaves, corolla 1 mm long, valvate, subglobose; stigma lobes not extending beyond disc.	*floribunda* (32)	Similar to *E.baroniana*.¹

## Supplementary materials

Supplementary material 1 Occurrences per identified Malagasy *Erica* species, plotted on elevation maps Data type pdf https://binary.pensoft.net/file/1334001This dataset is made available under the Open Database License (http://opendatacommons.org/licenses/odbl/1.0/). The Open Database License (ODbL) is a license agreement intended to allow users to freely share, modify, and use this Dataset while maintaining this same freedom for others, provided that the original source and author(s) are credited.Jan Hackel, Andriantsilavo H. I. Razafimanantsoa, Vincent Porcher, Michael D. Pirie

Supplementary material 2 Specimen voucher information for new DNA sequence data, including International Sequence Database Collaboration accession numbers Data type csv https://binary.pensoft.net/file/1334002This dataset is made available under the Open Database License (http://opendatacommons.org/licenses/odbl/1.0/). The Open Database License (ODbL) is a license agreement intended to allow users to freely share, modify, and use this Dataset while maintaining this same freedom for others, provided that the original source and author(s) are credited.Jan Hackel, Andriantsilavo H. I. Razafimanantsoa, Vincent Porcher, Michael D. Pirie

Supplementary material 3 Information on Erica pollen abundance in sediment chronosequences extracted from the literature Data type xlsx https://binary.pensoft.net/file/1334003This dataset is made available under the Open Database License (http://opendatacommons.org/licenses/odbl/1.0/). The Open Database License (ODbL) is a license agreement intended to allow users to freely share, modify, and use this Dataset while maintaining this same freedom for others, provided that the original source and author(s) are credited.Jan Hackel, Andriantsilavo H. I. Razafimanantsoa, Vincent Porcher, Michael D. Pirie
