# Supplementary figures and images for: ﻿Heathers (Erica, Ericaceae) of Madagascar: taxonomy, evolution, ecology and uses
Source: PhytoKeys. 2025 May 20;256:91–118. doi: 10.3897/phytokeys.256.141375 (PMC12117345; doi:10.3897/phytokeys.256.141375)

Fig. S1 – records per species

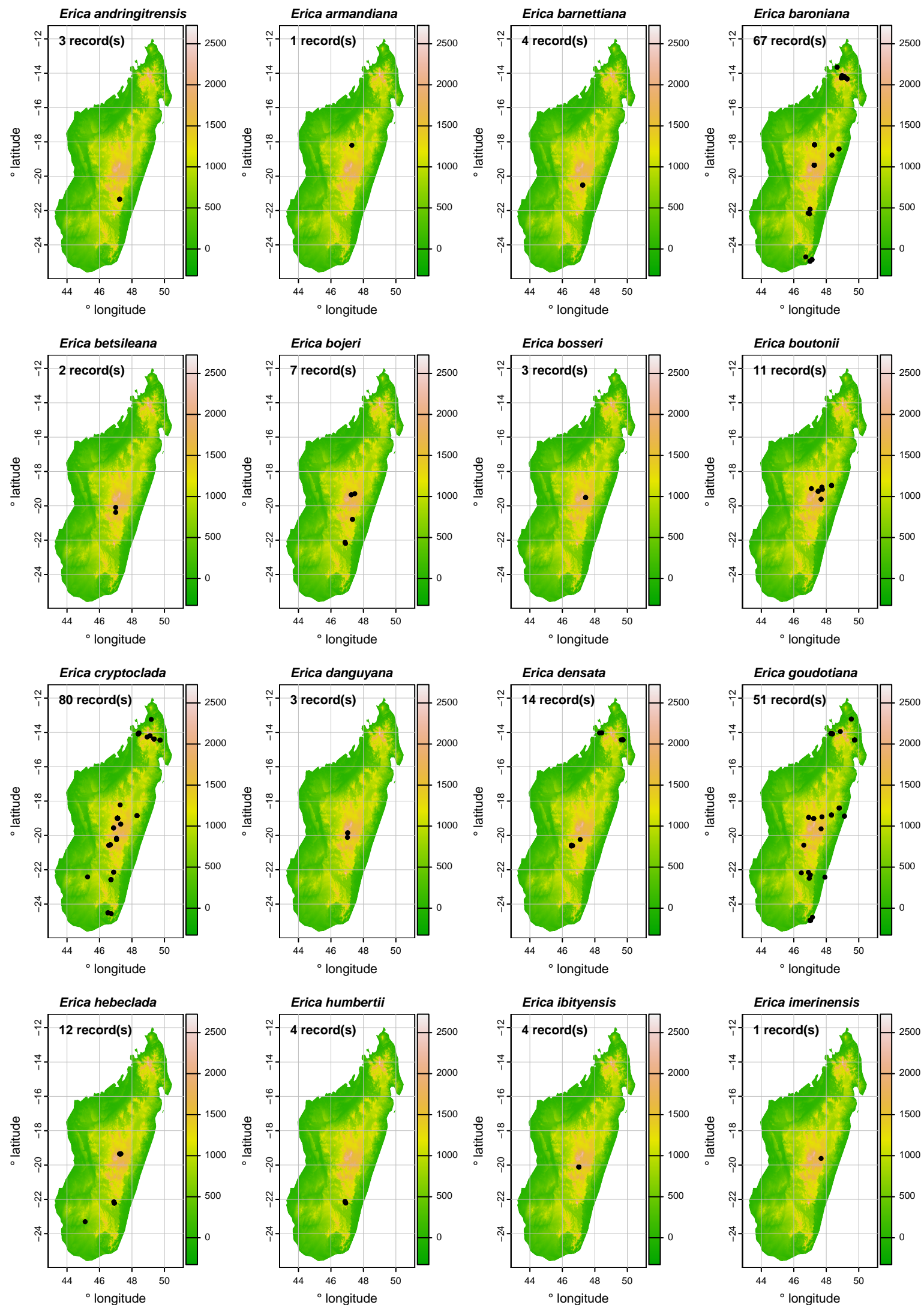

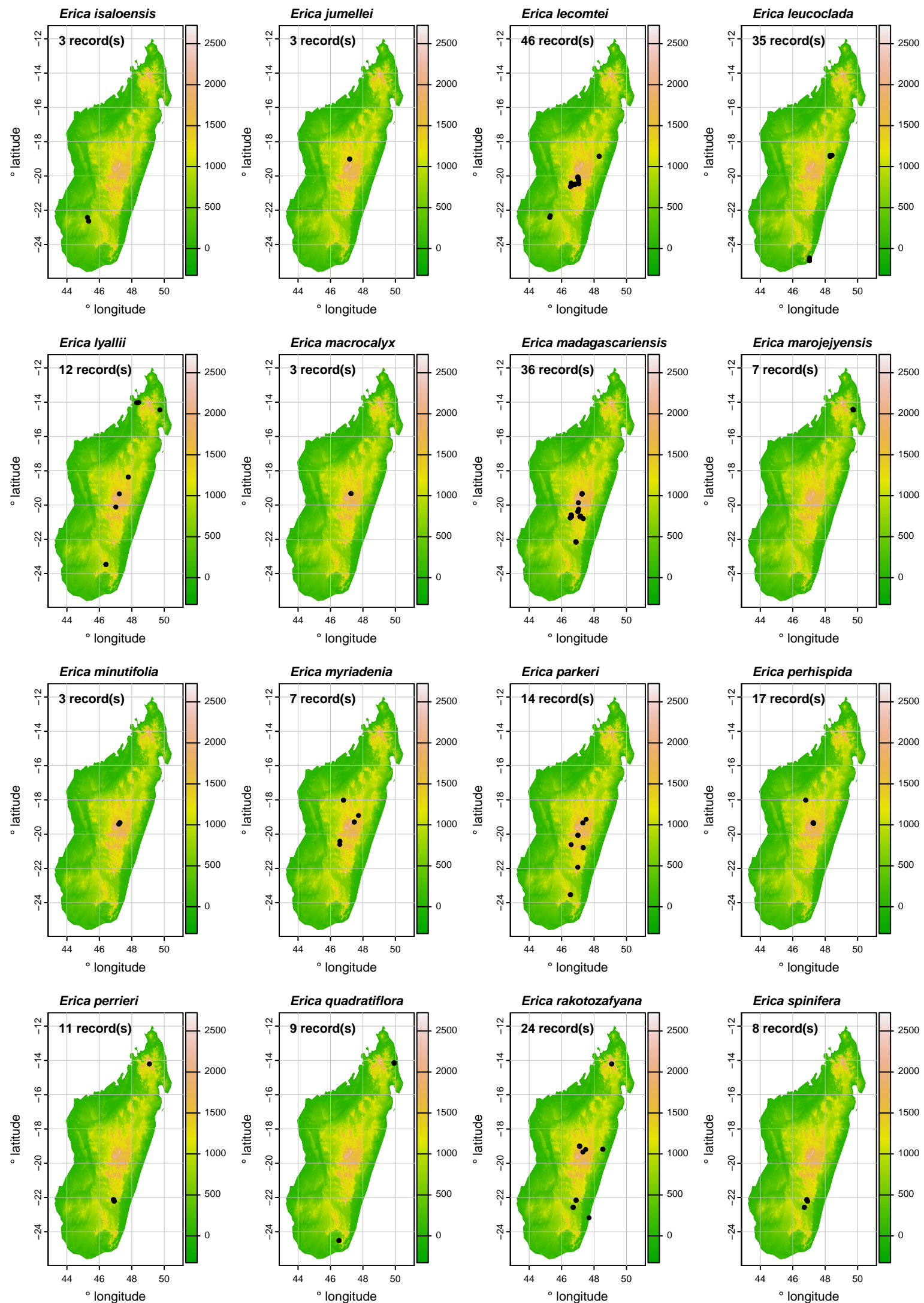

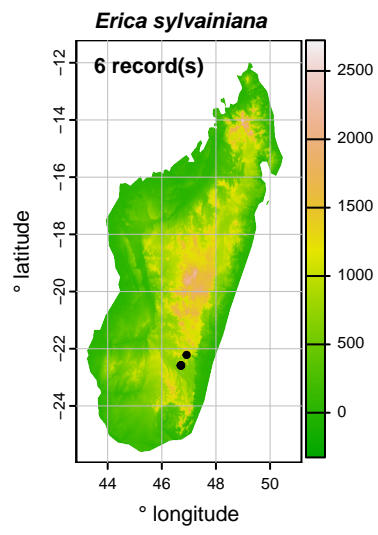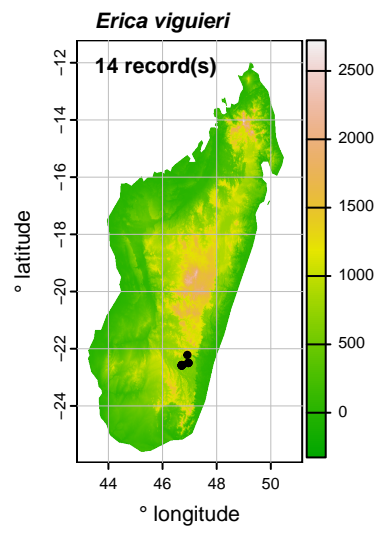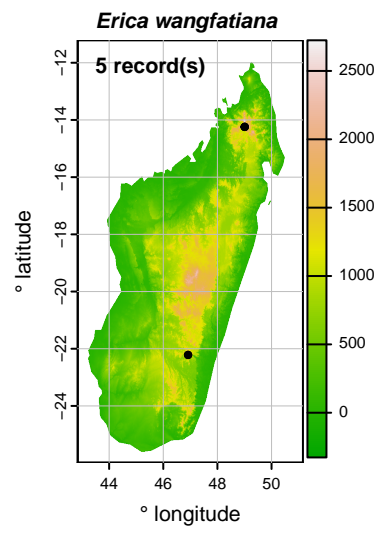

Supplement: Supplementary material 1 — Occurrences per identified Malagasy Erica species, plotted on elevation maps [file phytokeys-256-091_article-141375__-s001.pdf]
